# Improving shear bond strength of metallic brackets after whitening

**DOI:** 10.1590/2177-6709.25.5.038-043.oar

**Published:** 2020

**Authors:** Juan Fernando Aristizábal, Angela Patricia Polanía González, James A. McNamara

**Affiliations:** 1 Universidad del Valle, Department of Orthodontics (Cali, Colombia).; 2 Hamdan Bin Mohammed College of Dental Medicine (Dubai, UAE).; 3 University of Michigan, Department of Orthodontics and Pediatric Dentistry, School of Dentistry (Ann Arbor/MI, USA).

**Keywords:** Orthodontics, Tooth bleaching, Shear bond strength, Hydrogen peroxide, Sodium ascorbate

## Abstract

**Objective:**

To evaluate a protocol for bonding metallic brackets after bleaching with hydrogen peroxide (HP).

**Methods:**

60 extracted maxillary premolar were randomly divided into an unbleached control group and two groups bleached with a solution of 35% hydrogen peroxide prior to bonding. The teeth in one of the treated groups were bonded immediately after bleaching; while the other group was treated with 10% sodium ascorbate immediately after bleaching and before bonding. The teeth in all groups were stored in an artificial saliva solution for 7 days after bonding. The shear bond strength data was measured in megapascals (MPa) and the fail attempts were verified. The significance level was established at *p*< 0.05.

**Results:**

The unbleached group, in which brackets were bonded to untreated enamel, had the highest bond strength values (11.0 ± 5.7MPa) in comparison to the bleached group (7.14 ± 40MPa), in which brackets were bonded to recently bleached enamel. Slightly improved bond strength was observed in the antioxidant group (8.13 ± 5.4MPa), in which the teeth were bleached and then the antioxidant was applied to the teeth before bonding. Unbleached and bleached groups showed statistically significant difference for shear bond strength (*p*=0.03) and load strength (*p*=0.03); no significant differences were noted between unbleached and antioxidant groups (*p*=0.52).

**Conclusion:**

The antioxidant treatment applied immediately after bleaching was effective in reversing the reduction in shear bond strength of brackets after tooth bleaching.

## RESUMO

**Objetivo:**

Avaliar um protocolo para colagem de braquetes metálicos após clareamento com peróxido de hidrogênio (PH).

**Método:**

Sessenta pré-molares superiores extraídos foram aleatoriamente divididos em um Grupo Controle (sem clareamento) e dois grupos que receberam clareamento, antes da colagem, com solução de peróxido de hidrogênio a 35%. Em um dos grupos tratados (Grupo Clareamento), os braquetes foram colados imediatamente após o clareamento, enquanto o outro (Grupo Antioxidante) foi tratado com ascorbato de sódio a 10%, imediatamente após o clareamento e antes da colagem. Os dentes, em todos os grupos, foram armazenados em saliva artificial por 7 dias após a colagem. As informações relativas à resistência da colagem foram registradas em megapascals (MPa) e as tentativas fracassadas foram verificadas. O nível de significância foi estabelecido em *p* < 0,05.

**Resultados:**

O Grupo Controle, no qual os braquetes foram colados ao esmalte não clareado, apresentou os valores mais altos de força de adesão (11,0 ± 5,7 MPa), em comparação ao Grupo Clareamento (7,14 ± 40 MPa), no qual os braquetes foram colados ao esmalte recém-clareado. Uma suave melhora na força de adesão foi observada no Grupo Antioxidante (8,13 ± 5,4 MPa), no qual, após os dentes serem clareados, foi aplicado antioxidante aos dentes, antes da colagem. Os Grupos Controle e Clareamento apresentaram uma diferença estatisticamente significativa para a resistência da colagem (*p* =0,03) e a resistência à carga (*p*= 0,03); porém, nenhuma diferença significativa foi notada entre os Grupos Controle e Antioxidante (*p* =0,52).

**Conclusão:**

O tratamento antioxidante aplicado imediatamente após o clareamento foi efetivo em reverter a diminuição da resistência da colagem dos braquetes após o clareamento dentário.

## INTRODUCTION

The increasing popularity of tooth whitening/bleaching is having a considerably impact in smile design and dentistry.^1^ Many whitening systems are used to bleach enamel, some of them have concentrated solutions of hydrogen peroxide (HP), which is the most commonly used agent for whitening discolored teeth. These solutions are subjected to either heat or light to accelerate the bleaching reaction.^2^

Hydrogen peroxide can penetrate tooth structure for adequate stain removal. This penetration is effective due to the low molecular weight of hydrogen peroxide and its ability to denature proteins. This capability increases the tissue permeability allowing ions to move through the teeth.^3^ Hydrogen peroxide releases free oxygen radicals that lead to some cellular changes.^4^

Continuous whitening for at least two to four weeks has been shown to achieve a significant difference in bond strength. Nevertheless, some patients that have had their teeth previously bleached, often become more aware of orthodontic problems and want to be treated.^5^

To defeat clinical matters associated to compromised bond strength of teeth following bleaching, several techniques have been suggested. For example, Barghi and Godwin^6^ pre-treated bleached enamel with alcohol, while Kalili et al.^7^ and Sung et al.^8^ recommended to use adhesives containing organic solvents. It is often recommended, however, to postpone any bonding procedure until the whitening sessions are finished, due to the transitory decrease of bond strength found in freshly bleached enamel.^9^ ^-^ ^11^ It is recommended a waiting period of 24 hours up to four weeks for bonding procedures after bleaching.^11^ ^-^ ^16^ Decreased bond strength in bleached enamel has been associated to the inhibition of polymerization of resin-based materials as regards to the presence of residual oxygen.

Considering all the reported interactions that impact the bond strength of composite on bleached enamel,^17^ the present research arises as to whether there is any protocol that can be used to defeat the detrimental effects of bleaching on enamel. Thus, the purpose of this *in vitro* study was to evaluate a bonding protocol of metallic brackets after whitening with hydrogen peroxide. The null hypothesis is that there would be no significant differences in shear bond strength between the unbleached group and the antioxidant group.

## MATERIAL AND METHODS

### Preparation of specimens

This experimental *in vitro* study was approved by the institutional ethical committee of *Universidad del Valle* (#018-07). Total sample of 60 human maxillary premolars extracted for orthodontic purposes were collected and stored in saline solution that was changed every three days. The criteria for tooth selection included intact buccal enamel with no cracks caused by the extraction forceps, no caries, and no pretreatment with any chemicals. 

The samples were randomly divided into three equal groups of teeth, as follows (confidence interval set at 95%): A control group and two groups bleached with a 35% solution of hydrogen peroxide. Unbleached group (n=20) served as a control group; bleached group consisted of specimens bonded immediately after treated with hydrogen peroxide (n=20), while Antioxidant group specimens (n=20) were treated with a 10% solution of sodium ascorbate agent just before bonding and immediately after bleaching with the same agent used in bleached group. Sodium ascorbate is a form of vitamin C that can be used as an antioxidant and as an acidity regulator.

### Bleaching procedures

A commercial 35% solution of hydrogen peroxide in the form of an in-office bleaching gel (Pola Office®, SDI Limited, Victoria, Australia) was applied to the enamel surfaces of the embedded teeth from the bleached and antioxidant groups for two cycles of 20 minutes each, according to the manufacturer’s instructions.

### Application of antioxidant

After whitening procedure, the teeth from the antioxidant group were treated as follows: 10 ml of 10% sodium ascorbate was dripped on the enamel surfaces of the teeth and agitated with a sterile brush. After 15 minutes, the enamel was washed with distilled water and dried. 

### Bonding of brackets

Next step was to bond brackets to the buccal surfaces of all premolar in each group. Sixty identical stainless steel Orthos brackets (Ormco^®^ Corporation, Orange, CA) with an 0.022 x 0.028-in slot were used in this study. Right and left first maxillary premolars brackets with a bracket pad surface area of 12.95 mm² were used. 

The brackets were bonded to specimens at 24°C room temperature. Before composite bonding, all specimens were conditioned with a 37% phosphoric acid gel (3M Unitek, Monrovia, CA) for 30 seconds, then rinsed with water for 10 seconds and dried. The bonding primer used for all groups was Orthosolo^®^ (Ormco Corporation, Orange, CA), which has alcohol in its composition. 

For all the groups, the brackets were bonded with Enlight^®^ bonding system (Ormco Corporation), according to the manufacturer’s instructions. This composite was used, instead of others, because is the one that is routinely used in authors’ clinic. After the bracket was properly positioned on the tooth, each bracket was subject to 300g of force^18^ measured with a pressure dynamometer, and excess bonding resin was removed with a sharp scaler. The composite was light-cured for 10 seconds with a LED system (Ultralume 5, Ultradent, South Jordan, Utah) at a distance of 1 cm from the bracket. 

The teeth then were embedded in acrylic placed in phenolic rings (Veracryl^®^, New Stetic, Medellín, Colombia). A wood mounting jig was used to position the rings so that the facial surfaces of the teeth were positioned perpendicular to the bottom of the mold. The labial surfaces were oriented parallel to the applied force during the shear test.

### Artificial saliva immersion

Immediately after the bonding process, the specimens from all three groups were immersed in 250 ml of artificial saliva solution (Salivar^®^, Farpag Laboratories, Bogota, Colombia) at 37°C for 7 days. The artificial saliva solution had an electrolyte composition similar to the human saliva. After the specimens were removed from the artificial saliva, the enamel surfaces were rinsed with an air/water syringe for 30 seconds before the shear bond test. 

### Analysis of shear bond strength

The shear bond strength of the samples was measured with an Instron™ tensile testing machine (Instron Co., Norwood, MA) that was programmed to measure a crosshead speed of 1 mm/min. An occlusogingival oriented load was applied to the bracket, and produced a shear force at the bracket-tooth interface. A computer connected with the test machine recorded the results of each test in megapascals (MPa). 

### Statistical analysis

Comparisons of means were made with Student’s *t*-test and a Kaplan-Meier estimator to verify the failure time of the shear bond strength between the groups. The failures between the groups were analyzed with a Cox test. Both experimental groups were compared to each other and with the Control Group.

All statistical analyses were performed with Excel^®^ and with Stat^®^ software package (version 8.0, Stata Corporation, College Station, Texas). Significance for all statistical test was predetermined at *p*< 0.05.

## RESULTS

Descriptive statistics were computed for all analyzed variables and described as mean and standard deviation (SD) or as median and interquartile range (Table 1).

**Table 1:** Descriptive statistics for the shear bond strength of the three groups.

Variable	Group A (Control)	Group B (Bleached)	Group C (Antioxidant)	P*	P**	P***
Displacement						
Mean±SD	0,54±0,25	0,62±0,32	0,51±0,39	0,8	0,35	0,37
(Iqr)	(0,39-0,59)	(0,34-0,87)	(0,27-0,84)			
Load						
Mean±SD	0,14±0,076	0,09±0,05	0,11±0,07	0,03	0,1	0,52
(Iqr)	(0,06-0,18)	(0,05-0,13)	(0,05-0,15)			
Tensile						
Mean±SD	11±5,7	7,14±4	8,13±5,4	0,03	0,1	0,52
(Iqr)	(4,8-13,8)	(4,16-10,28)	(3,7-11,3)			

Comparisons between: *Group A x B, **Group A x C, ***Group B x C (in MPa). *p* = 0.05; SD = standard deviation; Iqr = interquartile range.

**Figure 1:**
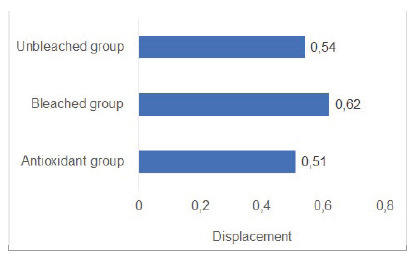
Displacement for each group.

**Figure 2:**
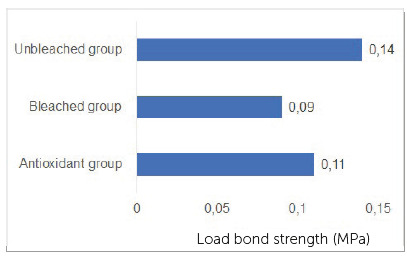
Load bond strength (MPa) for each group.

**Figure 3:**
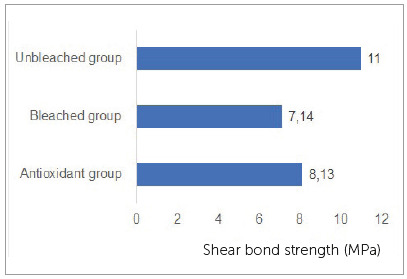
Shear bond strength (MPa) for each group.

The results for the Student *t*-test indicated no significant differences between Unbleached group and Antioxidant group, and between Bleached and Antioxidant groups, while comparisons between Unbleached and Bleached groups showed statistically significant difference for shear bond strength values and load strength values. Means for shear bond strength were: Unbleached group = 11.0±5.7; Bleached group = 7.14±4; Antioxidant group = 8.13±5.4 (Figs. 1, 2, 3).

**Figure 4:**
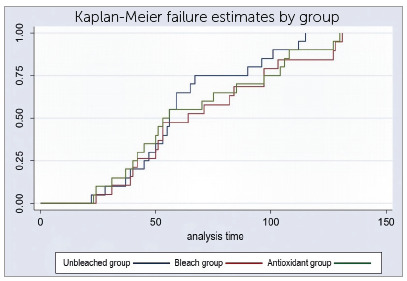
Kaplan-Meier failure estimates by group. P= 0.2.

The results of the Kaplan-Meier estimator indicated no significant differences between the groups; even though the control group showed a different behavior when a 0.75 MPa load was applied (Fig. 4). 

## DISCUSSION

The null hypothesis was accepted, since there were no significant differences between the unbleached group and the antioxidant group. This *in vitro* study determined a bonding protocol of metallic brackets after bleaching with hydrogen peroxide (HP). All groups were bonded with an alcohol-base bonding agent (Orthosolo^19^); Group C also was treated with sodium ascorbate after the bleaching process, in an attempt to restore the reduced shear bond strength of metal brackets, as suggested by Bulut et al^20^.

The present results indicate that sodium ascorbate applied to bleached enamel before immediate bonding with composite resin appeared to renew the decreased shear bond strength of metal brackets. This study also showed a reduction in shear bond strength of brackets post-bleaching (Bleached group), contrary to the control group (Unbleached group). Studies have shown that bond strength values required to withstand normal orthodontic forces are between 8 and 9 MPa.^21^

Some researchers have attempted to clarify the reduction in bond strength in enamel bleached with carbamide peroxide. Literature indicate that weak bonding surfaces and staining susceptibility are related to enamel surface morphology, with varying degrees of surface roughness and structural changes occurring through loss of prismatic formation.^22^ ^-^ ^24^

Previous investigations^9^ ^,^ ^11^ ^,^ ^12^ ^,^ ^15^ ^,^ ^23^ have indicated that *in vitro* immersion of specimens in artificial saliva, distilled water or even saline for at least 7 days showed a complete reversal of the reduced enamel bond strength. This process assumes that the immersion procedure removes residual oxygen from the bleaching material. Human saliva is supposed to have comparable action on the enamel after whitening.

Uysal et al.^16^ demonstrated the effect of 35% hydrogen peroxide bleaching agent on shear bond strength of metallic orthodontic brackets bonded to premolars immediately after whitening. This study concluded that immersing bleached teeth in artificial saliva does not have a significant effect on shear bond strength, but postponing bonding procedures for 2 to 3 weeks might be favorable. Uysal et al.^16^ agree with Bishara et al.,^25^ who have reported that immediate bond strength values were not affected adversely by 10% carbamide peroxide bleaching for a week. However, Miles et al.^11^ reported a significant decrease in bond strength of ceramic brackets after 72 hours of whitening with the same agent.

Josey et al.^23^ suggested that acid-etched bleached teeth have lost their regular prismatic boundaries, and such variations might affect the retentive qualities of dental restorations or adhesives applied to enamel surface. These investigators also reported that under experimental conditions, hydrogen peroxide diffuses out of the teeth from 1 to 6 weeks. 

To eliminate clinical effects associated to compromised bond strength post-whitening, Sung et al.^8^ suggested the use of adhesives containing organic solvents. They noted an interaction between bond strength to bleached enamel and the bonding agent used. Groups using Optibond™, an ethanol-based bonding agent, showed no significant reduction in bond strength between bleached and unbleached groups. However, All-Bond 2^®^ and One-Step^®^ bonding agents (Bisco Dental Products, Richmond BC, Canada) are acetone-based; these agents showed a significant decrease in bond strength between bleached specimens compared to unbleached controls. 

These observations agree with Kalili et al.^7^ regarding the differences in bond strength between various bonding agents, which may be associated to the presence of alcohol in the primer. They also mentioned that the application of an alcohol-based bonding agent can minimize the inhibitory effects of the whitening process by the interaction of alcohol with residual oxygen.

Bulut et al.^5^ demonstrated that bleaching of enamel with 10% CP immediately before bonding leads to a decrease of bracket tensile bond strength. They also showed that in samples to which antioxidant was dripped for 10 minutes immediately after whitening, tensile bond strength was found to be at the same level as in those samples kept just in artificial saliva solution after 7 days. 

Lai et al.^26^ also immersed the bleached specimens in 10 per cent sodium ascorbate solution for three hours. Their results showed that sodium ascorbate allows free-radical polymerization of the adhesive resin to proceed without premature termination by restoring the altered redox (reduction-oxidation) potential of the oxidized bonding substrate, thus reversing the compromised bonding. Khoroushi et al.^27^ and Kimyai et al.^28^ also suggest that shear bond strength can be restored after the application of an antioxidant in previous bleached teeth.

In the present study, three previously reported factors were used to eliminate the effect of whitening on shear bond strength of metal brackets: an ethanol-based bonding agent (Orthosolo^®^), the application of a sodium ascorbate solution for 15 minutes, and artificial saliva immersion for 7 days. According to the present results, those three factors can be used as a protocol to restore the effect of whitening in shear bond strength of metal brackets. Since this is an *in vitro* study, clinical bond-failure investigations are needed to validate the protocol performance proposed in this study. 

## CONCLUSIONS

1. The use of a 35% hydrogen peroxide in-office whitening system immediately before bonding reduces shear bond strength values.
2. Treating the bleached enamel surface with 10% sodium ascorbate reversed the decreased shear bond strength.
3. The use of an ethanol-based bonding agent with artificial saliva immersion during 7 days with the antioxidant may be an innovative option for fixed orthodontic treatment after whitening.
